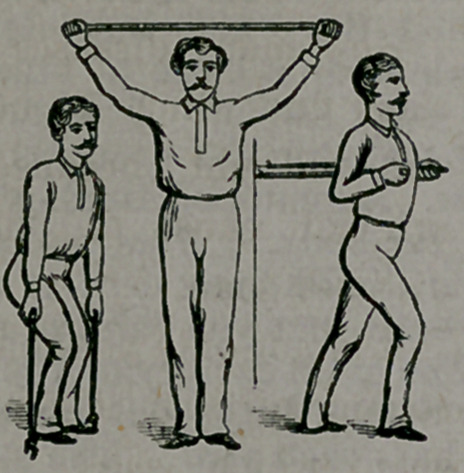# Health & Exercise

**Published:** 1875-10

**Authors:** 


### HEALTH AND EXERCISE.

We besought our friend Mr. Wood, of the Goodyear Rubber concern, to place on exhibition at the Fair his Pocket Gymnasium. We told him it would attract great attention, and win for him fame and fortune. But he found it out of his power to give the exhibition his personal attention, and so abandoned the idea.

But we have not changed our mind in regard to the attractive nature of such an exhibition of the capacity of his invention, as could be given. It is, beyond all question, we think, the best exercising arrangement to be found; and yet, like most excellent devices, it is simplicity itself.

It is merely a series of very elastic tubes, filled with plugs in either end, and having a controlling check attached. The series embraces seven tubes, of varying strength, and is designed to be used by all classes of people, from four years upwards. The smallest one costs a dollar, the largest two dollars. The heaviest tube will supply the strongest man with exercise to his heart's content. It is fitted with a screw eye and hook, to attach to the wall or floor. One of the best lifting-machines to be imagined, is thus secured at very small cost.

Now we have practised some with lifting-machines to very good purpose. But we have never been infatuated with them. We believe a set of these tubes, running from small to large, with screw-hooks set at various angles in the wall and floor and ceiling, would be far more useful to us and to a family. Those who attempt to *lift* every muscle of the body into strength and symmetry will be likely to fail.

But Mr. Wood's appliances leave nothing to be desired, as it seems to us. If chest or back or loins or arms need exercise, this system gives it in perfection. There are a multitude of graceful attitudes, difficult to assume without practice, which these tubes will enable the body to attain to.

We keep a set of the larger sized tubes in our sleeping apartment, and use them from time to time with the keenest satisfaction. We have a set in our editorial room, and get no little genuine inspiration from their use. If we are tired and cramped from long labor at the desk, we practice for three or four minutes with our little gymnasium, and our blood fairly bounds through our veins. We breathe deeper and freer for these occasional interludes of exercise. We can write better and faster after a few minutes of this exhilirating and enjoyable change.

Our professional friends ought to secure for themselves the benefits to be derived from this system of exercise. Doctors and ministers and lawers are too apt to neglect the salutary use of the muscles, which imparts activity to the circulation and tone to the system. They need some simple, handy means, for keeping the blood and the digestion active. This little gymnasium seems to furnish it.

The little cut which we insert here shows three of the many positions occupied in exercising with this device. Here is the lift, and here are shoulder and arm exercises. Hundreds of attitudes may be assumed, and nearly all the muscles, external and internal, are thus strengthened and vitalized.

The office for the sale of these goods is at Mr. Wood's rubber store, 697 Broadway, and here circulars explaining them and containing engraved representations of the tubes in use, may be had.

## Figures and Tables

**Figure f1:**